# Molecular Dynamics Analysis of Silica/PMMA Interface Shear Behavior

**DOI:** 10.3390/polym14051039

**Published:** 2022-03-04

**Authors:** Koochul Ji, Lauren K. Stewart, Chloe Arson

**Affiliations:** School of Civil and Environmental Engineering, Georgia Institute of Technology, Atlanta, GA 30332-0355, USA; kji9@gatech.edu (K.J.); lauren.stewart@ce.gatech.edu (L.K.S.)

**Keywords:** molecular dynamics, silica, interface, PMMA, shear strength

## Abstract

The mechanical properties of cementitious materials injected by epoxy have seldom been modeled quantitatively, and the atomic origin of the shear strength of polymer/concrete interfaces is still unknown. To understand the main parameters that affect crack filling and interface strength in mode II, we simulated polymethylmethacrylate (PMMA) injection and PMMA/silica interface shear deformation with Molecular Dynamics (MD). Injection simulation results indicate that the notch filling ratio increases with injection pressure (100 MPa–500 MPa) and temperature (200 K–400 K) and decreases with the chain length (4–16). Interface shear strength increases with the strain rate ($1\times 10^{8}$ s${}^{-1}$–$1\times 10^{9}$ s${}^{-1}$). Smooth interfaces have lower shear strengths than polymer alone, and under similar injection conditions, rough interfaces tend to be stronger than smooth ones. The shear strength of rough interfaces increases with the filling ratio and the length of the polymer chains; it is not significantly affected by temperatures under 400 K, but it drops dramatically when the temperature reaches 400 K, which corresponds to the PMMA melting temperature for the range of pressures tested. For the same injection work input, a higher interface shear strength can be achieved with the entanglement of long molecule chains rather than with asperity filling by short molecule chains. Overall, the mechanical work needed to break silica/PMMA interfaces in mode II is mainly contributed by van der Waals forces, but it is noted that interlocking forces play a critical role in interfaces created with long polymer chains, in which less non-bond energy is required to reach failure in comparison to an interface with the same shear strength created with shorter polymer chains. In general, rough interfaces with low filling ratios and long polymer chains perform better than rough interfaces with high filling ratios and short polymer chains, indicating that for the same injection work input, it is more efficient to use polymers with high polymerization.

## 1. Introduction

**Figure 1:**
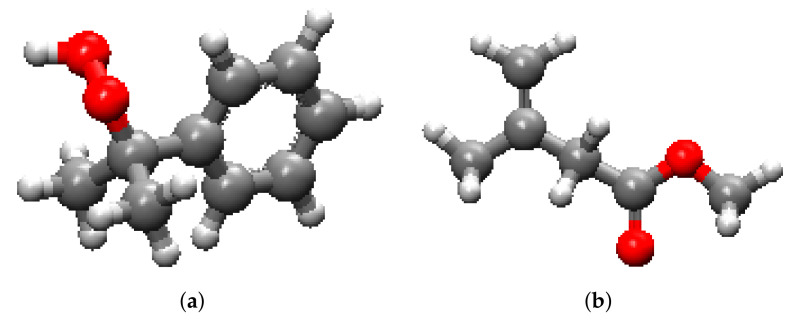
Molecular structures under study (**a**) Cumene hydroperoxide (CHP), (**b**) Methyl methacrylate (MMA-resin).

The use of sealants in repairing cracks in reinforced concrete components was originally designed to prevent the exposure of steel reinforcements to the atmosphere and subsequent alkali–silica reactions. As a result, most of the literature on the behavior of composites made of cementitious materials injected by epoxy focus on hydraulic properties (e.g., [1]). Recent studies (e.g., [2,3]) show that epoxies and polymethylmethacrylates (PMMAs) are the best-performing sealants: epoxies allow flexural strength to be recovered but can only be injected in larger cracks, while PMMAs, and in particular, High-Molecular-Weight Methacrylate (HMWM), can efficiently penetrate narrow cracks and reduce the permeability of concrete. It was recently found that steel-reinforced concrete members repaired by the gravity-driven deposition of HMWM exhibits higher resistance to bending than their non-repaired counterparts only if the crack width exceeds 0.1 mm [4]. To the best of the authors’ knowledge, the crack filling ratio expected when HMWM is applied under pressure is unknown, and the molecular origin of the flexural strength of repaired steel-reinforced concrete members is not understood. It was previously shown that the tensile strength of concrete/PMMA interfaces is due to van der Waals forces and that silica/PMMA interfaces exhibit higher tensile strengths than calcite/PMMA interfaces in mode I [5]. In this paper, we simulated PMMA injection and PMMA/silica interface shear deformation. Sensitivity analyses were conducted in order to understand the main parameters that affect crack filling and interface strength in mode II. In this study, of PMMA, different chain lengths were obtained by simulating curing processes that lead to different degrees of polymerization. Cross-linking represents the transition of PMMA to the glassy state, i.e., the process by which the hardener links with methyl methacrylate (MMA, i.e., resin) towards a cured state. Figure 1 shows the molecules of resin and hardener, and the principle of the crosslinking reaction is explained in [5]. We assumed that there was a free-radical polymerization of MMA. The radical initiator (cumene hydroperoxide) was decomposed to generate free radicals and to react with the MMA monomer [6]. The stoichiometric mixing ratio of methyl methacrylate and cumene hydroperoxide was 35:1, with a low initial density of 0.5 g/cm^3^.

Molecular Dynamics (MD) models have shed light on the mechanical behavior of numerous polymers and composite materials used to solve multi-physics problems [7,8,9,10,11,12,13,14]. The tensile deformation of bitumen/aggregate and polymer/mineral interfaces was recently investigated through molecular simulations [15,16,17,18]. MD was also used to understand the mode II behavior of interfaces between metallic atoms [19,20], polyethylene and silica [21] and carbon composites [22]. An MD model of glass with crystalline nano-precipitates revealed that glass/glass interfaces act as structural heterogeneities, which promote shear band formation and prevent strain localization [23]. MD was used to study the interfacial shear strength, structure and dynamics between calcium–silicate–hydrate (C-S-H) and polymer fibers and highlighted the importance of calcium atoms in the bond between the substrate and the fibers [24]. MD simulations allowed for the evaluation of the surface energy and interfacial adhesive properties of polymer/silica interfaces in various environmental conditions [25,26]. The influence of polymerization (i.e., polymer chain length) was highlighted in MD simulations of carbon nano-tube/polymer interface shear deformation [27].

According to the early works of Sasse and Fiebrich [28], the strength of bi-material interfaces is caused by mechanical interlocking induced by the percolation of one material into the voids of the other and by van der Waals forces. Interface entanglements were found to play a critical role in the strength of welded polymers [29]. Despite these important findings, the studies cited above did not investigate the interplay between interlocking and van der Waals forces. The aim of the present study, therefore, was to assess the pressure, temperature and PMMA chain length needed to fill a nano-scale crack by PMMA in a silica substrate, and to understand the influence of the resulting filling ratio on the shear strength of the silica/PMMA interface. Nano-imprint lithography (NIL) allows the production of repeatable nano-scale patterns and the injection of polymers in polycrystalline substrates. The relation between the molecular chain length and the mold cavity geometry was investigated in [30,31,32,33,34] to optimize pattern transfer. Inspired by these previous studies, we compared smooth and rough PMMA/silica interfaces, in which rough interfaces contained a notch of the order of 25 nano-meters in width and depth, typical of NIL processing. Section 2 describes the MD model of the interface between a silica substrate with a slit-shaped cavity and PMMA with different chain lengths. The results of PMMA shear deformation tests, MD notch injection tests and silica/PMMA interface shear deformation tests for different filling ratios are described and discussed in Section 3. Conclusions are summarized in Section 4.

## 2. MD Model Construction

**Figure 2:**
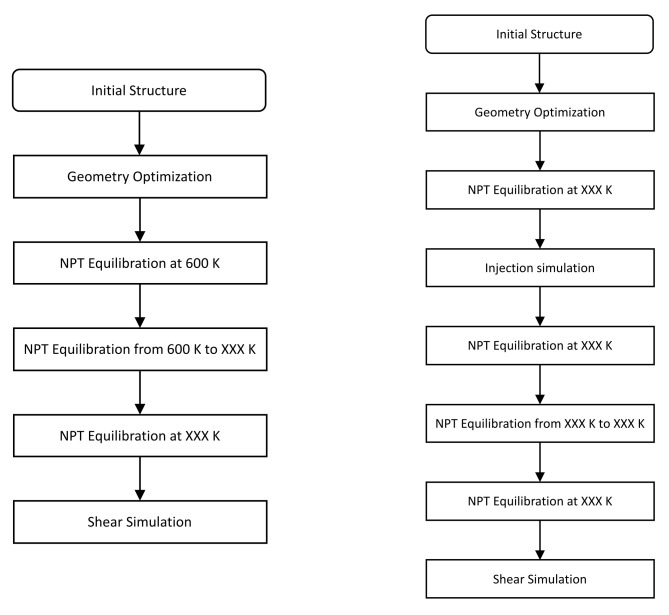
Main steps to build the smooth silica/PMMA interface (**left**) and the rough silica/PMMA interface (**right**).

The MD models were generated with MAPS 4.1. The model of silica substrate with a slit cavity model was created with the software VMD [35]. Figure 2 summarizes the main steps used to construct MD models of smooth and rough interfaces, and details on the protocol to generate the substrate/polymer interface are presented in the following section.

### 2.1. Force Field Equation

The choice of the force field equation is of primary importance in MD. Some researchers modeled interfaces between crystalline substrates and polymers with the Consistent Valence Force Field (CVFF) or the Polymer Consistent Force Field (PCFF) [36,37,38]. Here, we chose the DREIDING force field equation in all our simulations (cross-linking and polymerization, injection and shear deformation). The DREIDING force field was successfully employed for both organic and inorganic materials [21]. The reduced form of the DREIDING potential energy Equation [25,39] can be expressed as follows:

(1) $$\begin{array}{ccc} E_{t} & = & E_{b}+E_{nb} \end{array}$$

(2) $$\begin{array}{ccc} E_{b} & = & \sum_{b}\frac{1}{2}K_{b}{\left(b-b_{0}\right)}^{2}\;+\sum_{\theta }\frac{1}{2}K_{\theta }{\left(\theta -\theta_{0}\right)}^{2}\;+\sum_{\phi }K_{\phi }{\left(cos(\phi )\right)}^{i} \end{array}$$

(3) $$\begin{array}{ccc} E_{nb} & = & \sum_{i,j}4\epsilon_{ij}\left[{\left(\frac{r{*}_{ij}}{r_{ij}}\right)}^{12}-{\left(\frac{r{*}_{ij}}{r_{ij}}\right)}^{6}\right],\;r_{ij}\leq r_{c} \end{array}$$

In the second equation above, $E_{b}$ is the covalent bond energy, in which $K_{b}$, $K_{\theta }$ and $K_{\phi }$ are constants, and $b_{0}$ and $\theta_{0}$ are, respectively, the equilibrium bond length and bond angle. *b* is the bond stretch, θ is the bond angle and ϕ is the dihedral torsion angle. In the third equation above, $E_{nb}$ is the van der Waals energy (Lennard–Jones potential), in which $r_{ij}$ is the distance between the ith and jth atoms with charges $q_{i}$ and $q_{j}$. $r_{c}$ is the cutoff distance, equal to 12 Å in this study [17,39,40].

### 2.2. Smooth and Notched Silica Substrate

The mineral substrate was modeled with a lattice of silica cleaved in the [0, 0, 1] direction. The external dimensions of the silica box were 49.78×49.78×69.48Å^3^. This box was used as the model of a smooth silica/PMMA interface. Additionally, we built a model of substrate with a rectangular notch of dimensions 24.89×24.89×49.78Å^3^. Physically, the rectangular notch represented concrete surface roughness. Such a notch could be created with NIL in the laboratory. We used the substrate model with a notch to simulate rough silica/PMMA interface sliding tests.

### 2.3. MD Model of PMMA

**Figure 3:**
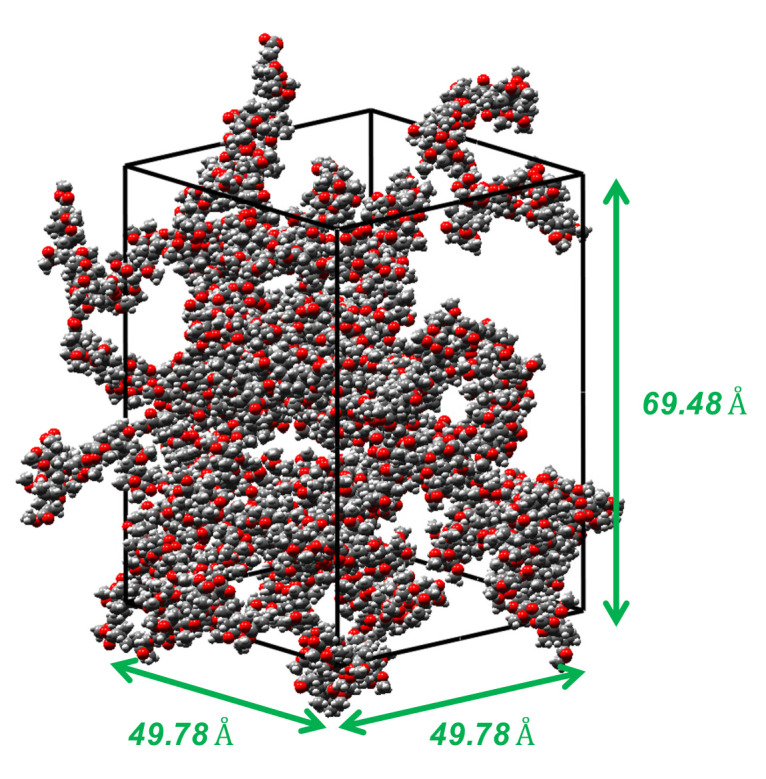
Amorphous cell composed of 50 PMMA chains of length 16.

PMMA is a cross-linked polymer which can exhibit different molecular chain lengths, depending on how cross-linking occurs. To study the influence of chain length on the mechanical behavior of the polymer and of silica/PMMA interfaces, we fabricated three PMMA models with isotactic polymethylmethacrylate chains that contained 4, 8 and 16 molecules with cumene hydroperoxide. The size of the PMMA substrate box was 49.78×49.78×69.48Å^3^. The details of each model are provided in Table 1. An amorphous cell of 50 polymer chains with a length of 16 is shown in Figure 3. We used the Verlet velocity algorithm to integrate the equations of motion. Temperature was controlled using the Nose–Hoover thermostat. Prior to conducting injection and sliding tests, equilibrium sequences were conducted to relax the high-energy configurations generated in the amorphous polymer structure. First, the energy of the polymer was minimized using the Conjugate Gradient algorithm, with a tolerance of $10^{-9}$. MD simulations were conducted with the NVT ensemble at 600 K, and the polymer structure was cooled to 300 K in 50,000 time steps (Δt=0.5 fs). Further relaxation was simulated for 50,000 time steps (Δt=0.5 fs) at 300 K to achieve the desired atomic structure under traction-free conditions.

## 3. Results and Discussion

All the shear simulations presented in this paper (for the polymer alone and for the interface systems) were conducted until failure was reached. Here, we define failure as the complete separation of the polymer or interface system into two sliding blocks, ductile behavior as behavior that exhibits multiple small stress drops before failure, and brittle behavior as behavior that exhibits a quasi-linear response followed by an abrupt stress drop at failure. We define shear strength as the maximum peak stress reached in the stress/strain curve. We applied a MATLAB smoothening function with a moving method to filter the raw results obtained by MD, which naturally present large fluctuations (see, for example, [39]). The peak stresses discussed below were calculated from those smoothed curves.

### 3.1. Simulation of PMMA Polymer Shear Deformation

The shear behavior of the PMMA polymers with chain lengths of 4, 8 and 16 was simulated with an NPT ensemble for various temperatures and strain rates, using the open-source Large-scale Atomic/Molecular Massively Parallel Simulator (LAMMPS). Cross-linking, polymerization and relaxation were simulated so as to achieve the mass density and curing temperature of PMMA. The calibration procedure of the model is explained in a previous study by the authors [5].

**Figure 4:**
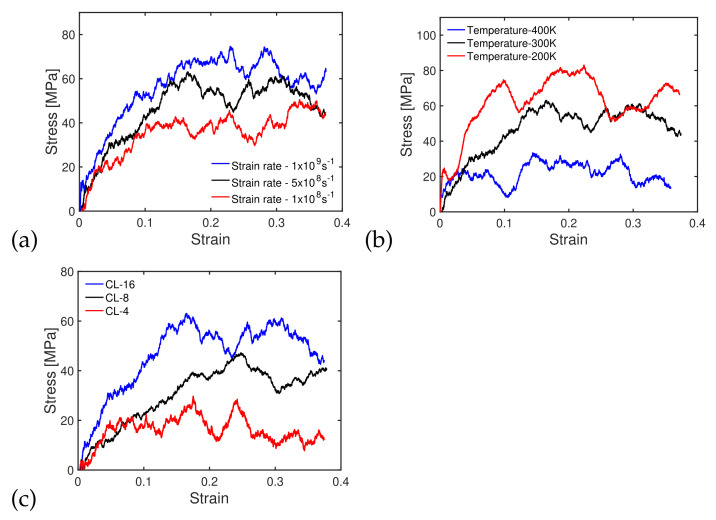
(**a**) Shear stress/strain curve of PMMA under different strain rates. Temperature: 300 K. Chain length: 16, (**b**) shear stress/strain curve of PMMA under different temperatures. Strain rate: $5\times 10^{8}$ s${}^{-1}$. Chain length: 16, (**c**) shear stress/strain curve of PMMA with different chain lengths. Strain rate: $5\times 10^{8}$ s${}^{-1}$. Temperature: 300 K.

Three different strain rates ($1\times 10^{8}$ s${}^{-1}$, $5\times 10^{8}$ s${}^{-1}$ and $1\times 10^{9}$ s${}^{-1}$) were applied to the polymer cell shown in Figure 3 at 300 K. The stress/strain curves obtained by MD simulation, plotted in Figure 4a, show that the strength (peak stress) of the polymer increased the strain rate, as also noted in previous studies [5,41]. In the following, we present simulations with a strain rate of $5\times 10^{8}$ s${}^{-1}$. We now compare the shear stress/strain curves of PMMA chains with lengths of 16 under temperatures of 200, 300 and 400 K. Figure 4b shows that the shear strength decreased as the temperature increased. This result is consistent with previous studies, which indicated that a higher temperature lowers the activation energy, reducing strength and elastic moduli [17]. The shear stress/strain curves of PMMA with chain lengths of 4, 8 and 16 under a temperature of 300 K and a sliding rate of $5\times 10^{8}$ s${}^{-1}$ are shown in Figure 4c. The behavior of the polymer was more ductile for shorter chain lengths and more brittle for longer chain lengths. The shear strength for a chain length of 16 (or 8 and 4, respectively) was of the order of 60 MPa (or 45 MPa and 25 MPa, respectively).

Although we did not dispose of any reference data at the nano-scale to calibrate the PMMA MD model for shear deformation, the model proposed here predicted behavior trends reported in the literature for similar materials or loading conditions [21,39,40]. Provided that the polymer model was already validated in a previous study [5], we thus considered that the PMMA model performed satisfactorily and could be used to build a silica/PMMA interface.

### 3.2. Simulation of PMMA Injection in a Notched Substrate and Measure of Filling Ratio

**Figure 5:**
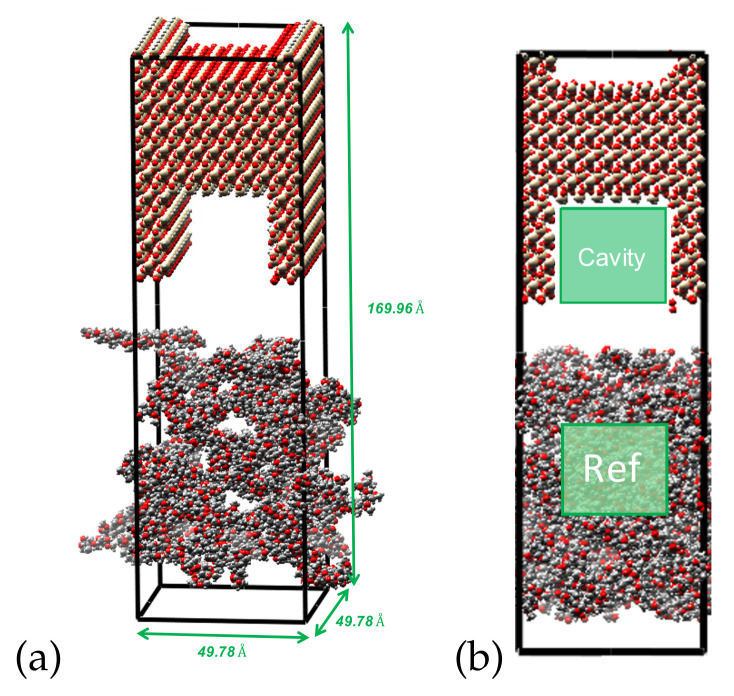
(**a**) Geometry of the interface model with a silica substrate that contains a slit cavity, (**b**) reference volume ($V_{reference}$) and cavity volume ($V_{cavity}$) to calculate the filling ratio.

To model the PMMA/silica interface system, we used the notched silica substrate and the overlay polymer. The dimensions of the interface system made of the notched silica and the polymer were 49.78×49.78×169.96${}^{3}$, as shown in Figure 5a. The potential energy of the PMMA system was first minimized with the NPT ensemble (the imposed number of particles, pressure and temperature) under 600 K for 50,000 time steps of Δt=1 fs. After the relaxation of the PMMA structure, the pressure was raised under a controlled temperature. The total injection period was 700 ps. The filling ratio F is defined as [30]:

(4) $$F=\frac{N_{cavity}/V_{cavity}}{N_{reference}/V_{reference}}$$

where $N_{cavity}$ is the number of atoms in the cavity of the silica substrate, $V_{cavity}$ is the volume of the cavity, $V_{reference}$ is a volume of reference in the polymer (see Figure 5b) and $N_{reference}$ is the number of atoms in that reference volume. We counted the number of atoms injected in the silica cavity to calculate the filling ratio under different injection conditions.

**Figure 6:**
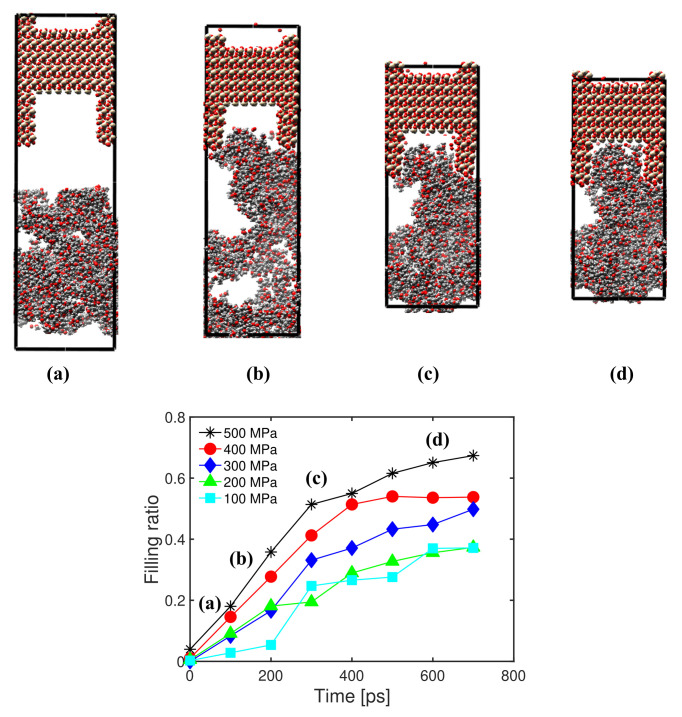
Evolution of the filling ratio over time under different injection pressures. Chain length: 16. Temperature: 300 K. Top: Snapshots of atomic configurations during the injection simulation for an injection pressure of 500 MPa.

In the following, we analyze the sensitivity of the filling ratio to the injection pressure and the chain length. After the equilibrium tests at 300 K, injection simulations were conducted with five different injection pressures (100, 200, 300, 400 and 500 MPa) under 300 K with a polymer chain length of 16. The simulation box was subjected to periodic boundary conditions on the lateral faces and to controlled pressure at the top and bottom faces. Snapshots of atomic configurations under an injection pressure of 500 MPa are shown in Figure 6. Over time, the length of the interface box decreased (because of the applied pressure), and the total number of atoms inside the slit increased. The curves shown in Figure 6 indicate that, as expected, the filling ratio increased when the injection pressures increased. The trend is clearer when comparing results at higher pressures.

**Figure 7:**
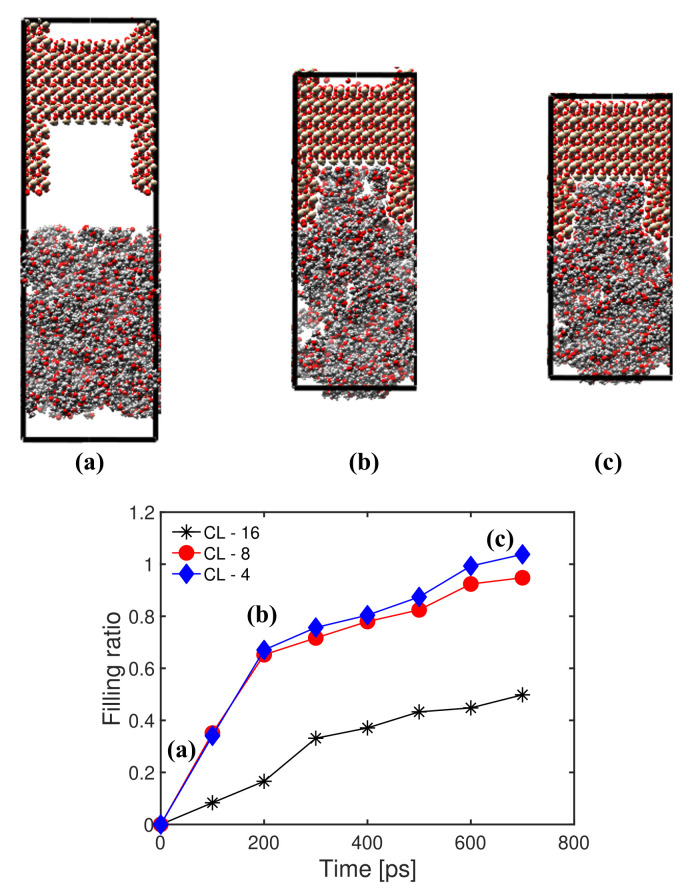
Evolution of the filling ratio over time for different PMMA chain lengths (CL). Temperature: 300 K. Injection pressure: 300 MPa. Top: Snapshots of atomic configurations during the injection simulation for a chain length of 4.

We now study the influence of PMMA chain length on the filling ratio for a temperature of 300 K and an injection pressure of 300 MPa. Figure 7 shows that the filling ratio decreased as the chain length increased. The trend was particularly noticeable as the chain length increased from 8 to 16, which can be explained by geometric compatibility. Indeed, the lengths of PMMA molecules of chain length 4, 8 and 16 were, without optimization, 10.1 Å, 20.2 Å and 40.5 Å, respectively. That means that the 4-mers and 8-mers could fit in the silica cavity (24.89 Å × 24.89 Å × 49.78 Å), but the 16-mers could only fit in the direction of the thickness of the model (see Figure 5a). The smaller difference in the filling ratio between the 4-mers and 8-mers can be attributed to the higher number of non-connected void spaces created by the entanglement of 8-mer chains. Snapshots of atomic configurations during the MD injection simulation with a chain length of 4 (Figure 7) clearly show the difference of cavity filling compared to the case simulated at the same temperature with a chain length of 16 and an injection pressure of 500 MPa (Figure 6).

**Figure 8:**
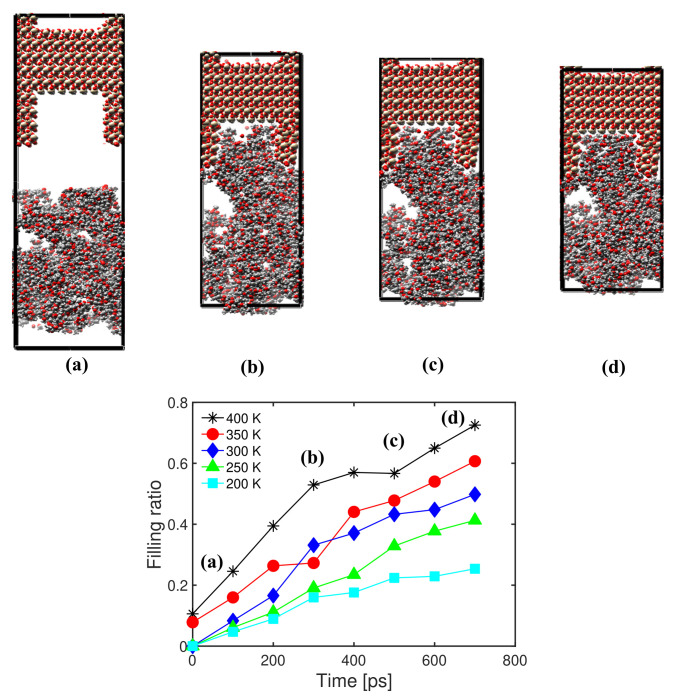
Evolution of the filling ratio over time under different temperatures. Chain length: 16. Injection pressure: 300 MPa. Top: Snapshots of atomic configurations during the injection simulation for a temperature of 400 K.

The results of simulations under five different temperatures (200, 250, 300, 350 and 400 K) for an injection pressure of 300 MPa and a chain length of 16 are plotted in Figure 8. In general, the higher the temperature, the higher the filling ratio. A jump in the filling ratio was observed when switching from 350 K to 400 K, because PMMA begins to melt in that temperature range. This trend is confirmed by the comparison of Figure 8 (at 400 K) and Figure 6 (at 300 K). For the range of parameters tested, the filling ratio was more sensitive to the temperature of injection than the injection pressure (see Figure 6 and Figure 8).

### 3.3. Simulation of Interface Shear Deformation

**Figure 9:**
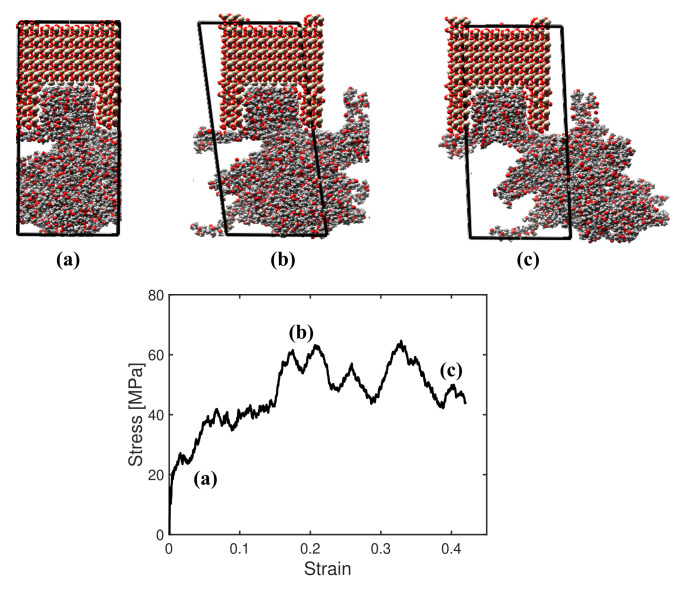
Shear test conducted on a PMMA/silica interface created with an injection pressure of 500 MPa under a temperature of 300 K and with a chain length of 16. The initial filling ratio (before the shear displacement was applied) was 68%. Note: the position of the black box does not represent the actual position of the box—it only shows the average displacement and strain fields of the box.

In the following, we compare interface shear tests with and without a notch in the substrate. The simulation box was free of stress in the lateral directions. The top layer of the mineral substrate was fixed (zero displacement), and the bottom face was subjected to shear strain at controlled rate. Figure 9 summarizes results obtained for a rough interface system with a filling ratio of 68% and a PMMA chain length of 16, generated by an injection pressure of 500 MPa under a temperature of 300 K. The first snapshot (a) was obtained during the elastic response of the material for shear strains below 0.1%. The subsequent snapshot (b) shows that damage developed in the polymer as the strain increased. The last snapshot (c) illustrates the failure of the interface. Interestingly, failure occurred in the polymer alone, and no silica/PMMA interface debonding was observed. This is a completely different response compared to that observed in mode I, which shows interfacial failure between polymer and minerals [5].

#### 3.3.1. Effect of Loading Conditions and Chain Length on the Interface Shear Strength

**Figure 10:**
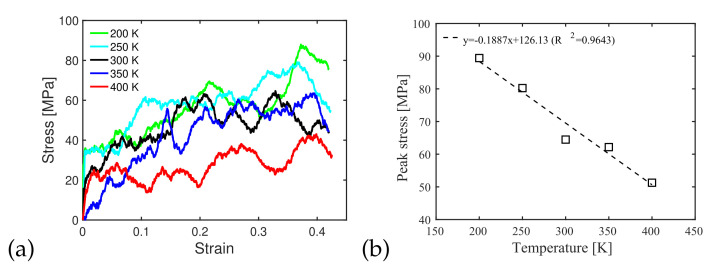
(**a**) Shear stress/strain curves of silica/PMMA interfaces subjected to a strain rate of $5\times 10^{8}$ s${}^{-1}$ under various temperatures. Chain length: 16. Injection pressure of 500 MPa. Injection temperature: 300 K. Filling ratio before shear test: 68%, (**b**) peak shear stress as a function of temperature during the interface shear tests conducted at a rate of $5\times 10^{8}$ s${}^{-1}$. Chain length: 16. Injection pressure of 500 MPa. Injection temperature: 300 K. Filling ratio before shear test: 68%.

We conducted interface shear tests under different temperatures and strain rates to compare the shear behavior of notched silica/PMMA interfaces created with PMMA molecules with chain lengths of 16, under an injection pressure of 500 MPa and an injection temperature of 300 K, for which, the filling ratio at a temperature below the melting point of PMMA was the highest among all the cases studied in Section 3.2 (68%, see Figure 6). Figure 10a shows that at a given strain, the shear stress generated by the imposed lateral displacement rate decreased dramatically when the temperature dropped from 350 to 400 K, which is the melting temperature of PMMA. The sensitivity analysis suggests a linear relationship between the peak shear stress and temperature for the range of parameters tested (Figure 10b).

**Figure 11:**
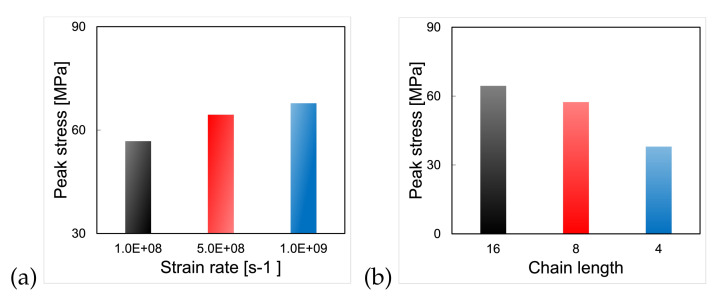
(**a**) Interface shear strength under different strain rates. Chain length: 16. Temperature: 300 K. Injection pressure of 500 MPa. Injection temperature: 300 K. Filling ratio before shear test: 68%, (**b**) interface shear strength for different chain lengths. Strain rate: $5\times 10^{8}$ s${}^{-1}$. Temperature: 300 K. Injection pressure: 500 MPa. Injection temperature: 300 K. Filling ratio before shear test: 70% (CL = 4), 67% (CL = 8), 68% (CL = 16).

The interface shear strength was extracted from the results of interface shear tests conducted at strain rates of $1\times 10^{8}$ s${}^{-1}$, $5\times 10^{8}$ s${}^{-1}$ and $1\times 10^{9}$ s${}^{-1}$ under a temperature of 300 K. The results (Figure 11a) show an increase in interface shear strength with the strain rate, consistent with the increase in PMMA shear strength with the shear strain rate (see Section 3.1). The influence of PMMA chain length on interface shear strength is illustrated in Figure 11b for a shear test conducted under a strain rate of $5\times 10^{8}$ s${}^{-1}$ and a temperature of 300 K. For each chain length considered, the injection pressure was 500 MPa and the injection temperature was 300 K, leading to pre-shear filling ratios of 70%, 67% and 68% for chain lengths of 4, 8 and 16, respectively. In the conditions tested, the longer the chain, the higher the interface shear strength. For shorter chain lengths, failure initiated in the polymer, away from the interface. As the chain length became longer (16), failure tended to occur closer to the polymer/silica substrate interface. This observation implies that, for the same injection work input, a higher interface shear strength can be achieved with entanglement (of long molecules chains) rather than with asperity filling (by short molecule chains). Note, however, that the simulations would have to be repeated to confirm this result, since the stress/strain curve likely depends on the initial state.

#### 3.3.2. Effect of Interface Roughness and Filling Ratio

**Figure 12:**
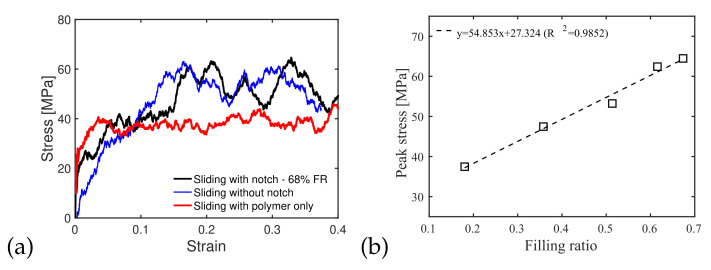
(**a**) Shear stress/strain curves obtained for the PMMA/silica interfaces with notch and without notch, and for PMMA alone. CL = 16. Sliding rate: $5\times 10^{8}$ s${}^{-1}$. Temperature during sliding: 300 K. Pre-shear filling ratio of the notched interface: 68 % (obtained under an injection pressure of 500 MPa and an injection temperature of 300 K, for a chain length of 16), (**b**) effect of filling ratio on the shear strength of the notched interface. Chain length: 16. Sliding rate: $5\times 10^{8}$ s${}^{-1}$. Temperature during sliding: 300 K. Pre-shear filling ratios of 18%, 36%, 51%, 61% and 68% were obtained with injection pressures ranging from 300 MPa to 500 MPa.

We now compare the shear behavior of interfaces with and without a notch to the shear behavior of the PMMA polymer alone. Simulations were conducted with a chain length of 16 and a temperature of 300 K. Interfaces with a notch had a pre-shear filling ratio of 68% (injection pressure: 500 MPa; temperature of injection: 300 K). The results are presented in Figure 12. Interestingly, the stress/strain curve of the rough interface was similar to that of the PMMA alone, which indicates that the strength of the interface was mostly contributed to by the PMMA chains. For both interface models (with and without a notch), simulation snapshots show that cracks initiated within the polymer and that major failure occurred in the vicinity of the interface. Failure occurred at a lower strain for the interface without a notch, and the shear strength of the interface with a notch was higher than that of the interface without a notch. Note that the plots obtained in Figure 12 were obtained for one replicate of each case. The simulations would need to be repeated to assess the effect of the initial state.

Peak shear stresses (shear strengths) were extracted from the results of interface shear tests conducted under a temperature of 300 K and a strain rate of $5\times 10^{8}$ s${}^{-1}$ with a chain length of 16, for five initial filling ratios (18%, 36%, 51%, 61% and 68%) obtained under different injection conditions. Figure 12b shows that the interface shear strength increased linearly with the filling ratio. Interestingly, a similar shear strength was obtained for an interface generated under 300 K with a 500 MPa injection pressure with molecules with chain lengths of 4 (see Figure 11b) and for an interface generated under 300 K with a 300 MPa injection pressure with molecules with chain lengths of 16 (see Figure 12b, filling ratio of 36%). These qualitative trends seem to indicate that stronger interfaces could be built with less mechanical work input if the PMMA presents higher polymerization, but this observation needs to be confirmed by repeating the simulations with several initial states. Since the strength of the polymer increased with the chain length (see Figure 4), it is recommended to use longer PMMA chain lengths for concrete repair, even if the filling ratio may not be as high as that for shorter chain lengths under the same injection conditions. This could be accomplished by adjusting the polymerization process to generate longer polymer chains, e.g., by changing the mixing ratio of methyl methacrylate and cumene hydroperoxide and/or the temperature of the mix during polymerization. Smooth silica/PMMA interfaces fail at lower shear strain more than PMMA alone or rough interfaces, indicating that the silica/PMMA bonds are the weak link of the interfaces. As a result, concrete reparation with PMMA injection will perform better if crack faces present asperities. We hypothesize that the reaction forces between the PMMA and the substrate at the cavity wall are the main factors that makes an interface with a notch stronger than an interface without a notch. Rough interfaces fail first in the polymer at low polymerization, even with a high filling ratio. This means that at low polymerization, the strength of a rough interface is limited by the polymer strength (and not by the PMMA/silica bonding forces). It is noted that when the PMMA presents high polymerization, the rough interface fails in the silica substrate. The benefit of using long PMMA chains is three-fold: (i) the polymer exhibits higher shear strength for higher polymerization; (ii) the entanglement of long chains increases the shear resistance of rough interfaces, helping the transfer of failure from the PMMA to the silica; (iii) for the same injection work input, interfaces with long PMMA chains perform better (i.e., they have a higher shear strength and reach failure at a higher shear strain).

#### 3.3.3. Potential Energy Evolution

**Figure 13:**
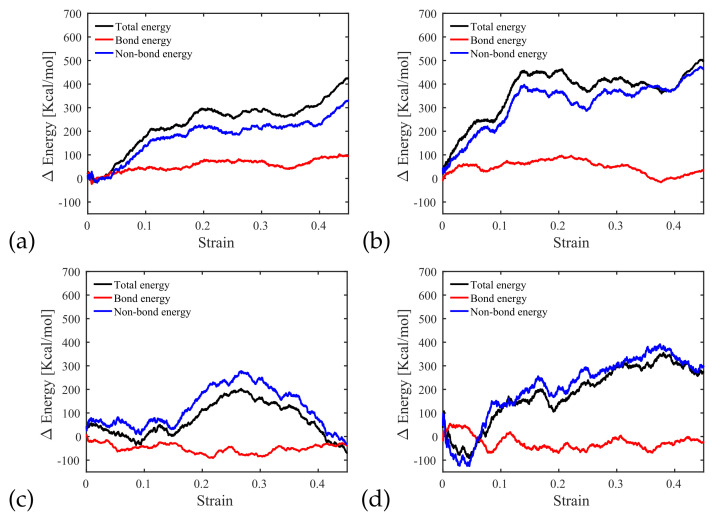
(**a**) Energy distribution during the sliding test of an interface without notch for chain length 16, (**b**) energy distribution during the sliding test of an interface with notch for filling ratio 68% and chain length 16, (**c**) energy distribution during the sliding test of an interface with notch for filling ratio 18% and chain length 16, (**d**) energy distribution during the sliding test of an interface with notch for filling ratio 68% and chain length 4.

**Figure 14:**
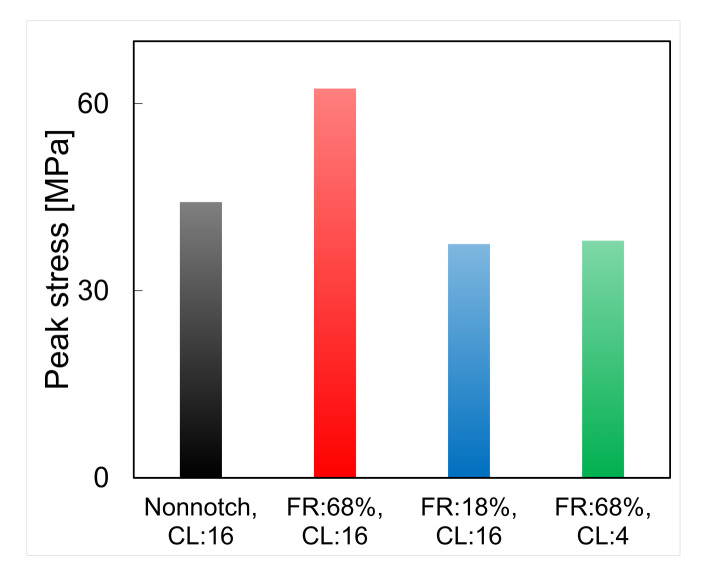
Effect of presence of notch, filling ratio, chain length on the shear strength.

In the following, we examine the distribution of energy that contributes to the work carried out towards interface shear failure. For a chain length of 16 and a filling ratio of 68%, the energy of interfaces with and without a notch is at 85% to 95% by the contribution made by van der Waals forces (Figure 13a,b). In other words, both types of interface mostly store non-bond energy, which is in agreement with previous studies [21,39]. Comparing Figure 13b (chain length of 16, filling ratio of 68%) and Figure 13c (chain length of 16, filling ratio of 18%), we observe that van der Waals forces alone provide more resistance to shear than the overall DREIDING force field, and bonding forces work towards shear failure (due to attraction interactions between atoms). A similar observation can be made by comparing Figure 13b (chain length of 16, filling ratio of 68%) and Figure 13d (chain length of 4, filling ratio of 68%), where less van der Waals energy is engaged in the mechanical work that leads to shear failure for shorter chain lengths. Interestingly, similar resistance to shear is obtained for an interface with a notch filled at an 18% filling ratio with polymer chains with lengths of 16 and for an interface with a notch filled at a 68% filling ratio with polymer chains with lengths of 4 (see Figure 14). However, less non-bond energy is developed in the specimen with a low filling ratio and long chains (Figure 13c) than for that with a high filling ratio and short chains (Figure 13d), which indicates that interlocking plays a critical role in shear resistance in interfaces with long polymer chains. This conclusion is supported by the fact that the failure of notched interfaces filled with long polymer chains (at any filling ratio) occurs in the substrate, close to the interface, while interfaces filled with short polymer chains (at a high filling ratio) fail in the PMMA, away from the interface.

## 4. Conclusions

In this study, we modeled a notched silica/injected PMMA interface system with MD to investigate the performance of concrete reparation via PMMA injection. Injection simulation results indicate that the notch filling ratio increases with injection pressure (100 MPa–500 MPa) and temperature (200 K–400 K) and decreases with the chain length (4–16). During the MD interface sliding tests, the shear stress increased monotonically with strain until the curves exhibited a pseudo-plateau at the onset of damage. The shear strength increased with the strain rate ($1\times 10^{8}$ s${}^{-1}$–$1\times 10^{9}$ s${}^{-1}$). Smooth interfaces were shown to have a lower shear strength than the polymer alone, and under similar injection conditions, rough interfaces tended to be stronger than smooth ones, indicating that concrete reparation via PMMA injection will perform better for crack faces that present asperities. The shear strength of rough interfaces increased with the filling ratio and the length of the polymer chains; it was not significantly affected by temperatures under 400 K, but it dropped dramatically when the temperature reached 400 K, which corresponds to the PMMA melting temperature for the range of pressures tested. For the same injection work input, a higher interface shear strength can be achieved with the entanglement of long molecule chains rather than with asperity filling by short molecule chains. Overall, the mechanical work needed to break silica/PMMA interfaces in mode II was mainly contributed by van der Waals forces, but it is noted that interlocking forces played a critical role in interfaces created with long polymer chains, in which, less non-bond energy was required to reach failure in comparison to an interface of the same shear strength created with shorter polymer chains. When the interface shear resistance was due to van der Waals forces (typically, for short chains and high filling ratios), failure occurred in the polymer, away from the interface. When the interface shear resistance was due to interlocking forces (typically, for long chains at any filling ratio), failure occurred in the substrate, close to the interface. In general, rough interfaces with low filling ratios and long polymer chains performed better than rough interfaces with high filling ratios and short polymer chains, indicating that for the same injection work input, it is more efficient to use polymers that present high polymerization. We hypothesize that the reaction forces between the PMMA and the substrate at the cavity wall are the main factors that makes an interface with a notch stronger than an interface without notch. The shear strength of smooth interfaces was limited by the PMMA/silica bond strength. At low polymerization, the strength of a rough interface was limited by the polymer strength. The benefit of using long PMMA chains is three-fold: (i) the polymer exhibits higher shear strength for higher polymerization; (ii) the entanglement of long chains increases the shear resistance of rough interfaces, helping the transfer of failure from the PMMA to the silica; (iii) for the same injection work input, rough interfaces with long PMMA chains perform better (i.e., they reach failure at higher shear strain and exhibit a higher shear strength). The results presented in this paper are based on a single instantiation of each interface system. Future work will be dedicated to the effect of the initial state, chain length and interface system size.

## Figures and Tables

**Table 1:** Characteristics of the MD models of PMMA.

Model Name	Chain Length	Nb. of Atoms in Each Chain	Nb. of Chains	Total Nb. of Atoms
CL-4	4	62	200	12,400
CL-8	8	124	100	12,400
CL-16	16	248	50	12,400

## Data Availability

This study did not report any data.
